# Endovascular Treatment of Large Vessel Occlusion Strokes Caused by Infective Endocarditis: A Systematic Review, Meta-Analysis, and Case Presentation

**DOI:** 10.3390/life12122146

**Published:** 2022-12-19

**Authors:** Ashkan Mowla, Saeed Abdollahifard, Saman Sizdahkhani, Erfan Taherifard, Fatemeh Kheshti, Kasra Khatibi

**Affiliations:** 1 Division of Stroke and Endovascular Neurosurgery, Department of Neurological Surgery, Keck School of Medicine, University of Southern California (USC), Los Angeles, CA 90089, USA; 2 School of Medicine, Shiraz University of Medical Sciences, Shiraz P.O. Box 71348-14336, Iran; 3 MPH Department, Shiraz University of Medical Sciences, Shiraz P.O. Box 71348-14336, Iran

**Keywords:** infective endocarditis, large vessel occlusion, stroke, mechanical thrombectomy

## Abstract

Thromboembolic events such as acute ischemic strokes are frequently seen in patients with infective endocarditis (IE). It is generally recommended that the administration of intravenous thrombolytics is avoided in these patients as they might encounter a higher risk of intracranial hemorrhages. In this setting, particularly with a large vessel occlusion (LVO), a mechanical thrombectomy may be an alternative option. In this systematic review and meta-analysis, we aimed to investigate the outcomes and safety of mechanical thrombectomies for LVO stroke patients secondary to IE. A search strategy was developed and we searched PubMed, Scopus, Web of Sciences, and Embase using the words “infective endocarditis”, “stroke”, and “mechanical thrombectomy”. Including 6 studies and 120 patients overall, this study showed that a mechanical thrombectomy might reduce the National Institute of Health Stroke Scale (NIHSS), with a weighted mean difference of −3.06 and a 95% CI of −4.43 to −1.70. The pooled rate of symptomatic intracranial hemorrhages and all-cause mortality were also determined to be 15% (95% CI: 4–47%) and 34% (95% CI:14–61%), respectively. The results of this study showed that a mechanical thrombectomy might be an effective and reasonably safe option for the treatment of LVO strokes caused by IE. However, more large-scale studies are needed to consolidate these results.

## 1. Introduction

Thromboembolic events in the central nervous system (CNS) are frequently the presenting symptom in infective endocarditis (IE), although overall cerebral ischemic events secondary to infections are a rare condition [1,2,3]. Studies have demonstrated that higher rates of CNS embolic events are associated with *Staphylococcus aureus* IE when the anterior leaflet of the mitral valve is affected [4]. In terms of the pathophysiology of strokes in these patients, a thrombus may develop as a result of a bacterial proliferation or bacterial invasion of heart tissue such as the leaflets. These thrombi may reach the CNS and occlude vessels [5,6]. The middle cerebral artery (MCA) is the most common location of ischemic events, but multifocal or distal ischemic events are also seen [7]. CNS complications in IE such as an acute ischemic stroke (AIS) are associated with significant morbidity and mortality, and should be treated with antibiotics as soon as possible [2]. When patients present with AIS, the standard algorithm for the treatment generally includes the potential administration of an intravenous thrombolysis (IVT). In patients with IE, however, treatment with IVT should be more weighted due to the high risk of hemorrhagic complications [8,9]. Considering that stroke patients with IE have a higher mortality rate and a higher risk of an intracranial hemorrhage (ICH) than stroke patients without IE (23.5% vs. 6.5%), this recommendation would be more consolidated [10,11]. Consequently, if a large vessel occlusion (LVO) is confirmed in the setting of AIS, then patients should be evaluated for a mechanical thrombectomy (MT) [8]. Although there have yet to be randomized clinical trial studies to prove the outcomes of MT in the setting of IE, many reports have shown that it is a successful and safe treatment [8,12,13]. Here, we discuss a case of AIS with LVO secondary to IE, which was successfully treated with MT, utilizing a novel stent retriever. We then carried out a systematic review and meta-analysis on the safety and outcomes of this procedure as a treatment of strokes due to IE.

## 2. Materials and Methods

This study was carried out according to the Preferred Reporting Items for Systematic Reviews and Meta-Analyses (PRISMA) protocol [13].

In light of MeSH words and related articles, a comprehensive search strategy consisting of “infective endocarditis”, “stroke”, “mechanical thrombectomy”, and related words was developed (Supplementary Materials Part A). We included studies that investigated MT as a treatment for strokes due to IE. We omitted studies with fewer than four cases, editorials, case reports, comments, letters to the editor, and conference abstracts as well as studies with full texts in languages other than English.

PubMed, Scopus, Web of Sciences, and Embase were searched using this search strategy in September 2022. The yield of our search was imported into an Endnote library (Niles Software, Philadelphia, PA, USA) and the duplicates were omitted. Through a two-step screening process, the eligible articles were identified. In the first step, the titles and abstracts were checked; in the second step, full texts were explored to develop the final library of included articles. Data extraction was initiated on the screened articles to collect data regarding characteristics such as the names of authors, countries where the data were gathered, design of each study, number of patients, demographic indexes of the patients such as age and gender, and desirable outcomes including all-cause mortality, complications, and recurrence rates. In all these steps from screening to data collection, two independent researchers were involved. If any conflicts were raised, it was discussed with a third researcher. We used no automated tools except Rayyan, a web-based tool for conducting systematic reviews, which was utilized for the screening and blind reviewing of the articles [14]. Quality and risk of bias assessments were performed via the appropriate National Institute of Health (NIH) tool for quality assessments. Based on the design of the articles, NIH provided an explicit questionnaire for this task. The results of each assessment were good, fair, and poor. If the quality of an article was determined to be poor, we sought to omit it from our study.

R statistical software version 4.2.0 (R Core Team (2022), R Foundation for Statistical Computing, Vienna, Austria), and Comprehensive Meta-Analysis (CMA) (Biostat Incorporation, NJ, USA) were used to perform the analyses. Regarding the different settings and designs of the included studies, a random model was preferred over fixed models for the pooling of the effect sizes. As the primary data were rates of outcomes (rate of ICH, mortality, and recurrence of stroke) and changes in the mean (change in National Institute of Health Stroke Scale, NIHSS), the final effect sizes were reported as an estimated proportion and weighted mean differences (WMD) as well as their 95% confidence intervals (CI) in a forest plot, respectively. The heterogeneity in each analysis was explored by Higgins’s index (I^2^) and publication bias by Egger’s test and a funnel plot. If a publication bias was noted in an analysis, the trim and fill method was used to adjust the estimated point. The final effect sizes were interpreted using a 95% CI.

## 3. Results

Before proceeding to the results of the systematic review and meta-analysis, we present a related case that may be easier to follow for the readers.

### 3.1. Case Presentation

**Figure 1:**
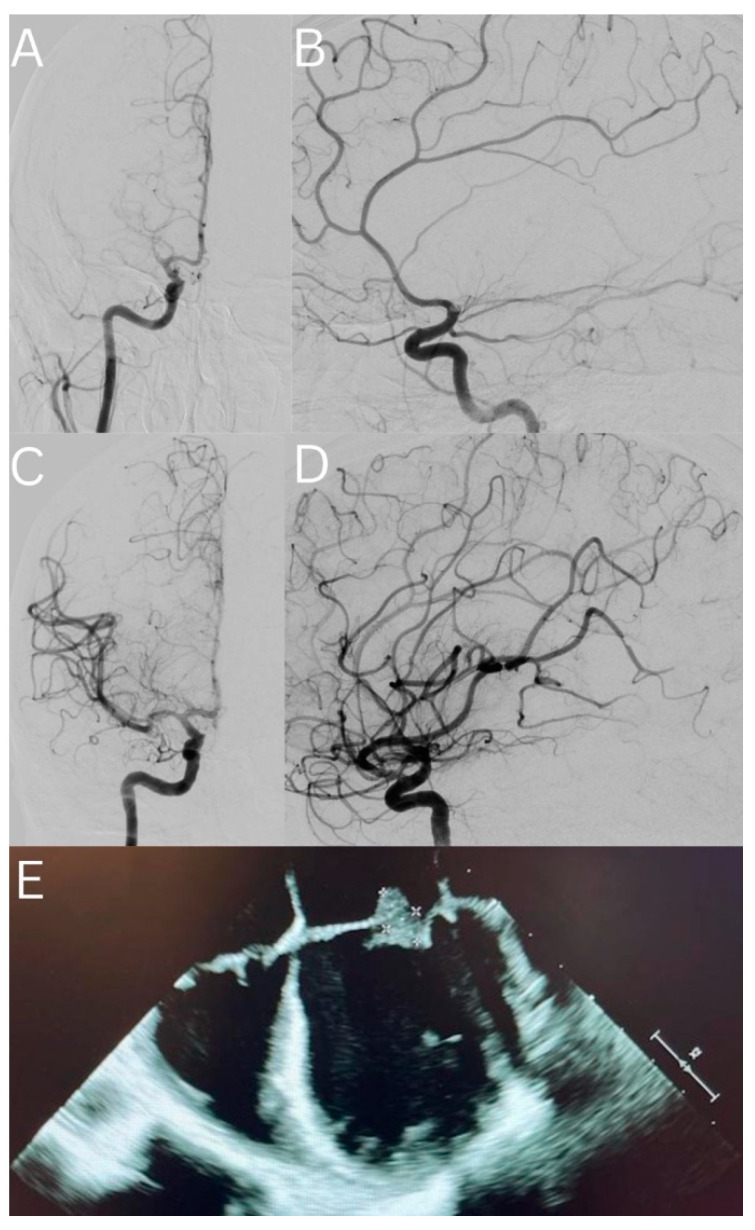
(**A**) Pre-op right internal carotid artery (ICA) angiogram (AP view), which shows right MCA (proximal M1 segment) occlusion; (**B**) pre-op right ICA angiogram (lateral view), which shows right MCA (proximal M1 segment) occlusion; (**C**) post-op right ICA angiogram (AP view), which shows complete re-canalization of the right MCA (TICI score of 3); (**D**) post-op right ICA angiogram (lateral view), which shows complete re-canalization of the right MCA (TICI score of 3); (**E**) transesophageal echocardiogram of the patient demonstrating a mobile vegetation on the mitral valve.

A thirty-year-old woman with a history of cocaine abuse presented with acute onset left hemiplegia, dysarthria, and a rightward gaze preference. Her NIHSS was 19 and presented 90 min after her last known well time. Head computed tomography (CT) and CT perfusion imaging demonstrated a large MCA distribution stroke and an Alberta Stroke Program Early CT Score (ASPECTS) of 10, with a significant perfusion mismatch of the right MCA territory. CT angiography (CTA) confirmed a proximal right M1 LVO. On the initial assessment, the patient was febrile to 40 °C with a high clinical suspicion of IE; therefore, IVT was not used. Given her markedly disabling neurologic deficits and the presence of a proximal LVO with an ideal perfusion mismatch profile, the decision was made to proceed with a cerebral angiogram and possible MT. The initial angiogram confirmed a proximal right M1 occlusion (Figure 1A–D).

The decision was made to perform MT, for which a CEREBASE DA (Cerenovus, Irvine, CA, USA) guide sheath, an LBC Aspiration catheter (Cerenovus, Irvine, CA, USA), and an Embotrap III with measurements of 5 mm × 37 mm (Cerenovus, Irvine, CA, USA) were used. One pull led to a successful opening of the vessel with a Thrombolysis in Cerebral Infarction (TICI) score of 3 (Figure 1). Standard post-thrombectomy care was initiated in the intensive care unit. Notable laboratory data from the admission included a leukocytosis of 39,000 (cells per microliter), a positive result for cocaine on the urine toxicology screen, and two sets of blood cultures growing oxacillin-susceptible *Staphylococcus aureus*. She had an unremarkable transthoracic echocardiogram, but her transesophageal echocardiogram demonstrated vegetative thickening within the atrial aspects of both the anterior and posterior mitral valve leaflets (Figure 1E). On hospital day two, magnetic resonance imaging of the brain demonstrated 4.8 cm of an acute infarct within the right basal ganglia and corona radiata with a confluent petechial hemorrhage and 4 mm of a right-to-left midline shift. Multiple small embolic infarcts were present throughout the bilateral cerebral and cerebellar hemispheres. On hospital day five, a CTA of the head did not show any evidence of mycotic aneurysms. An interval CT head scan on hospital day eight demonstrated evolving infarcts and stable petechial hemorrhages without evidence of a worsening mass effect or a new hemorrhage. The patient underwent a mitral valve replacement on hospital day nine. The patient was discharged to a rehabilitation facility with an NIHSS of two for mild left facial droop and mild left arm weakness; her degree of disability was measured as a modified Rankin Scale (mRS) of one.

### 3.2. Details of the Included Studies

**Figure 2:**
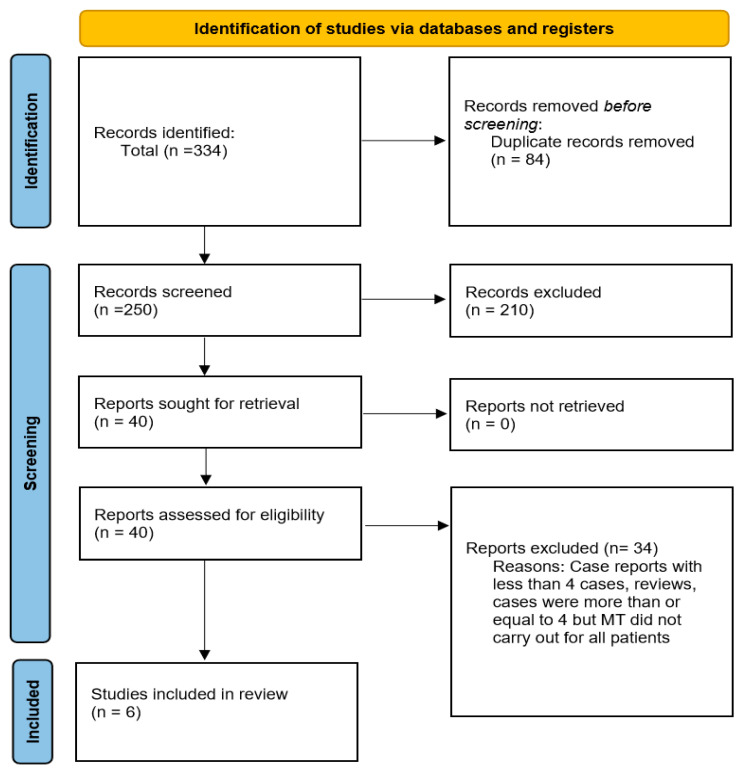
PRISMA flowchart of included studies.

Following the deletion of 84 articles from the primary 334 studies, 250 articles remained for screening; of these, 6 articles met our eligibility criteria and were included in this review. The details of the search and screening processes are illustrated in Figure 2. In total, 120 patients with a mean/median age range of 57.2 to 75.5 years were reviewed and included studies published between 2018 and 2021. Of those, 46 patients were female; from the reported microbes that tested positive for IE, *Enterococcus faecalis* and *Staphylococcus aureus* were the most common. In terms of risk factors, hypertension (HTN), hypercholesterinemia, and atrial fibrillation were more frequent. Overall, 25 patients received an intravenous thrombolysis (IVT) in combination with MT. The MCA (M1 segment) was reported to be the most common site of occlusions. ICHs were the most frequent complication, both periprocedural and in follow-ups, and MT resulted in a treatment in cerebral infarction (TICI) score of 2b and higher in 89 patients out of 120. The quality of the included studies was determined to be “good”, and no study was omitted (Table 1 and Table 2).

Feil et al. performed a case-control study on 55 patients with IE-related strokes and compared the results with matched patients with cardioembolic strokes who were all treated with MT using propensity score matching. Gathering data from the German stroke registry, they matched patients using age, gender, mRS, and NIHSS with a 1:2 ratio. In the first 24 h after the treatment visit, the mRS (median in IQR: 5 (95% CI of 4 to 5) vs. 4 (95% CI of 3 to 5), respectively) and NIHSS (16 (95% CI of 5 to 29) vs. 11 (95% CI of 4 to 18), respectively) of patients in the IE group were significantly higher than the controls. In terms of in-hospital complications, a recurrent stroke was the only complication that was significantly different between the two groups, and was reported to be higher in patients with IE (7 events (12.7%) in IE vs. 4 events in the controls (3.8%)). It is worth mentioning that the rate of ICH was not significantly different. In a longer follow-up visit, the outcomes of the cardioembolic patients were remarkably better, including mRS (3 (95% CI of 1 to 6) vs. 6 (95% CI of 3 to 6), a good functional outcome or mRS = 0–2 (43.3% vs. 20%), and mortality or mRS equal to six (28.8% vs. 60%). Using a univariate logistic regression, this study illustrated the variables that could be a determinant factor for a good outcome (mRS = 0–2) in the follow-up or mortality. These results demonstrated that endocarditis (odds ratio of 0.32 with a 95% CI of 0.11 to 0.87 for a good outcome and 4.49 with a 95% CI of 1.89 to 10.68 for mortality), age (odds ratio of 0.95 with a 95% CI of 0.91 to 0.98 for a good outcome and 1.05 with a 95% CI of 1.02 to 1.09 for mortality), and NIHSS (odds ratio of 0.88 with a 95% CI of 0.82 to 0.95 for a good outcome and 1.07 with a 95% CI of 1.02 to 1.13 for mortality) were identified as notable determinants in this analysis [15]. Another case-control study was carried out by Marnat et al., and matched IE patients treated with MT and patients with LVO that were related to atrial fibrillation (AF) and treated with MT. In line with the results of the previous study, a favorable outcome—which was defined as having an mRS of between 0 and 2—was significantly higher in the AF-related stroke patients (50.6% vs. 25.9%). The recurrence of strokes was also higher in the IE-related patients compared with the other group (25% vs. 0). Congruent with Feil et al., the rate of ICH was not significantly different between the patients [16]. In a case series of eight patients with IE-related strokes, Marquardt et al. included four patients treated with thrombolytics (LVO was not noted) and four patients who underwent MT due to IE-related LVO. Although these two groups were not comparable due to the different severity of diseases at the baseline and the type of stroke, the patients who underwent MT showed worse outcomes 24 h after the treatment, which could be due to the more severe condition of the MT patients at the baseline. In addition to reporting cases from their center, the authors carried out a systematic review of similar cases and compared 19 thrombolytic-treated patients with 21 MT-treated ones. The analysis of this study concluded that the rate of ICH was significantly higher in the first group (63% vs. 18%). No other outcomes, including mRS less than 2 (*p*-value = 0.20) and mortality at the time of admission (*p*-value = 1.00), were found to be significantly different between these two treatments [17]. A series of 15, 12, and 6 patients who were treated with MT due to IE-related strokes were reported by Sader et al. [18], Ramos et al. [13], and Ambrosioni et al. [19], respectively. All three studies reached a conclusion that MT may be an effective and safe treatment option, especially in IE-related LVOs where the administration of thrombolytics could increase the chance of ICH.

### 3.3. Results of the Meta-Analysis

**Figure 3:**
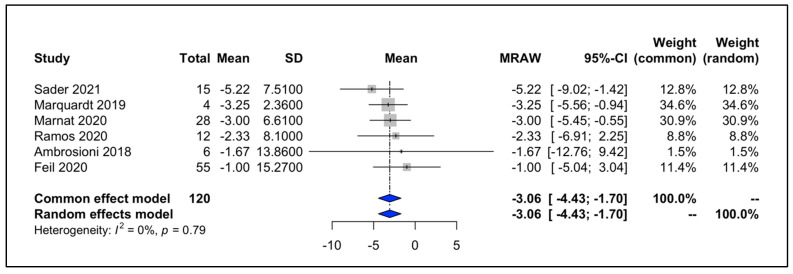
Forest plot of changes in NIHSS of patients after MT, [13,15,16,17,18,19].

**Figure 4:**
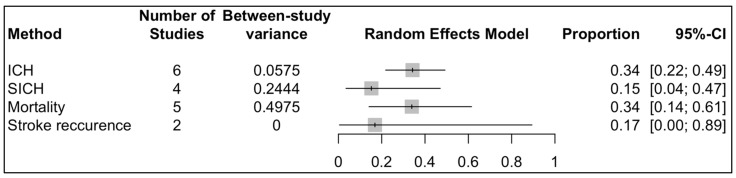
Forest plot of pooled rate of ICH, symptomatic ICH, all-cause mortality, and stroke recurrence.

**Figure 5:**
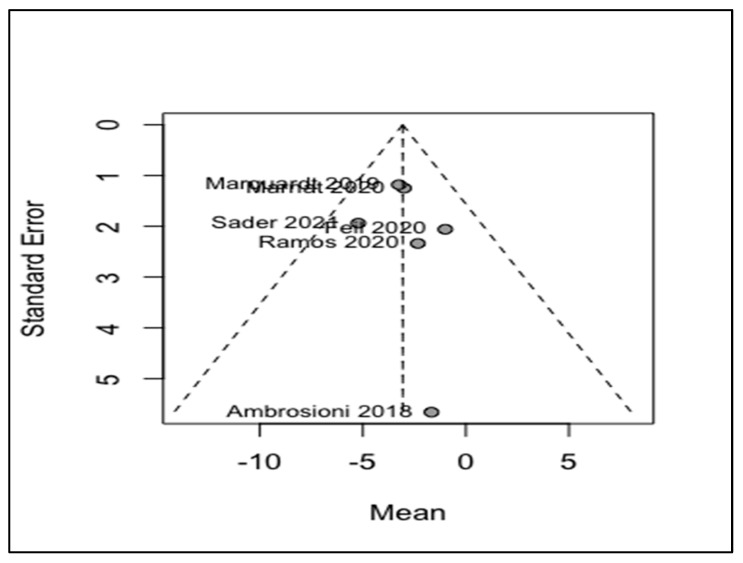
Funnel plot of pooled WMD of NIHSS, [13,16,17,18,19].

**Figure 6:**
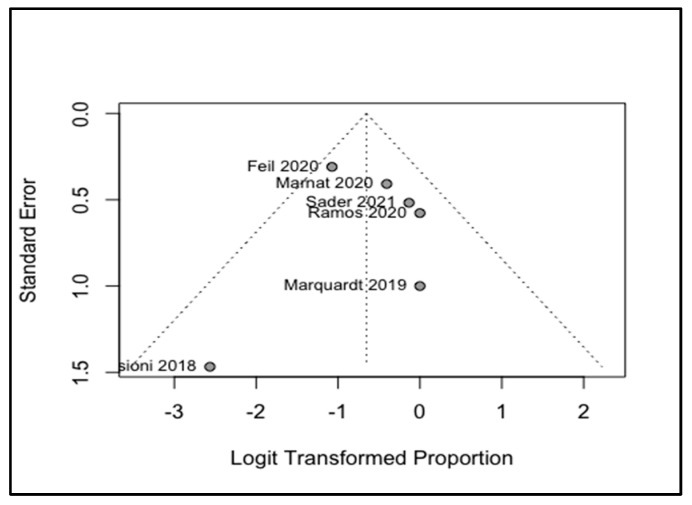
Funnel plot of pooled rate of ICH, [13,15,16,17,18,19].

**Figure 7:**
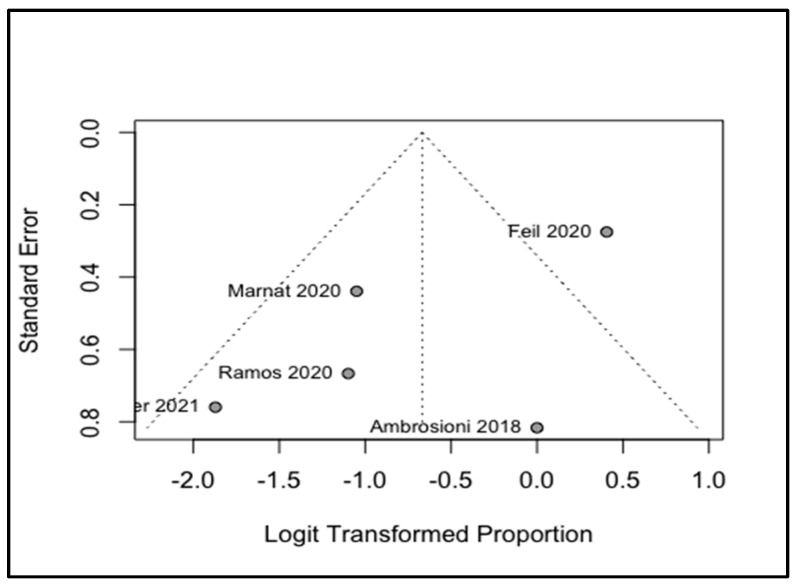
Funnel plot of pooled rate of all-cause mortality, [13,15,16,18,19].

Four analyses were conducted to investigate the following indices: changes in NIHSS, comparing before and after the treatments and the pooled rates of all-cause mortality; the occurrence of ICH; and the recurrence of strokes. Our study showed that MT could significantly reduce the NIHSS of the patients with a WMD of −3.06 and a 95% CI of −4.43 to −1.70 (Figure 3). The I^2^ was equal to zero and the results of the common and random model effects were the same. A total of 117 patients were included in the analysis of the rate of ICH, resulting in a pooled rate of 34% (95% CI: 22–49%) without any remarkable heterogeneity (I^2^ = 0; Figure 4). In terms of the rate of symptomatic ICH, the pooled rate was 15% (95% CI: 4–47%) with a low heterogeneity (I^2^ = 28%). By pooling the all-cause mortality rate of 5 studies and 116 patients, the overall mortality rate was determined to be 34%, with a 95% CI of 14%–61% and a remarkable heterogeneity (I^2^ = 74%). Only two studies investigated the recurrence of strokes, and a meta-analysis of these data led to an overall rate of 17% (95% CI: 0.00–89%) with an I^2^ of 48%. Although only six studies were used in this analysis, and Egger’s test might not be useful for the detection of publication bias, applying this test to the extracted data showed no remarkable publication bias. To cover the weakness of this test, funnel plots for the analysis of the NIHSS, ICH, and all-cause mortality were produced (Figure 5, Figure 6 and Figure 7; Supplementary Materials part B).

## 4. Discussion

In this systematic review and meta-analysis, a total of 6 studies were included that contained the details of 120 patients with AIS resulting from septic emboli originating from endocarditis treated with MT. The study showed that MT was associated with a statistically significant decrease in the NIHSS of these patients (WMD of −3.06 with 95% CI of −4.43 to −1.70). The analyses also showed that the pooled rates of post-thrombectomy ICH (any), symptomatic ICH, recurrence of AIS, and all-cause mortality were 34% (95% CI of 22% to 49%), 15% (95% CI of 4% to 47%), 17% (95% CI of 0.00 to 89%), and 34% (95% CI of 14% to 61%), respectively.

The American Heart Association/American Stroke Association guidelines recommend the administration of IVT such as alteplase for eligible patients with AIS as soon as possible [20,21]. However, due to the limited available body of evidence and higher rates of certain complications, there is no such consensus for patients with AIS originating from IE and IVT is not generally recommended for such patients [8]. In a nine-year retrospective study, data from 222 and 134,048 patients with AIS with and without IE were analyzed [9]. For all these patients, thrombolytics were used. It was shown that patients with IE had more than three times higher odds of ICH compared with patients without IE (adjusted odds ratio of 5 with a 95% CI of 2 to 12). The rate of favorable neurological outcomes upon discharge from the hospital was also lower in these patients (adjusted odds ratio of 0.1 with a 95% CI of 0.03 to 0.4). Consistently, the rate of mortality for acute LVO strokes estimated by our meta-analysis was more than double the rate reported in patients without IE. In a meta-analysis on 2233 patients with LVO, the rate of post-thrombectomy mortality was 14.9% [22].

This higher risk of ICH and unfavorable outcomes in patients with IE may stem from different pathological events in the course of emboli formation between them and patients without endocarditis. Virchow’s triad of stasis in the blood vessels, injuries to the cell linings of the vessels, and the presence of a hypercoagulability state are the main bases for thrombus formation [23]. However, a septic embolus originating from the vegetations of IE is more than just a composite of fibrin, blood cells, and glycoproteins; it is a thrombus containing microbial materials [24]. Therefore, the occlusion of a blood vessel and the following ischemia/necrosis are not the sole consequences of a septic embolus; it is an infectious embolus and could result in both vascular and infectious insults to the CNS [25]. In addition to the direct invasion of micro-organisms to the wall of the affected vessel, the microbial materials within the embolus could further lead to inflammatory and immune responses, the upregulation of their signaling pathways, and subsequent endotheliitis; therefore, the neurologic complications could include a wide range of pathological conditions in the form of meningitis, cerebritis, encephalitis, and abscess formation, etc. [11,26]. Mycotic aneurysms are also another complication that could develop secondary to septic embolization as well as the involvement of the wall of lamina blood vessels, which is accompanied by an increased risk of ICH [27]. As such, and to have a firm and clear therapeutic plan for these patients, further studies with higher levels of evidence and larger sample sizes are required.

Based on the literature, it appears that the risk of cerebral hemorrhagic complications is not only greater in those with AIS secondary to IE, but also the rate is higher in patients undergoing an infusion of thrombolytics compared with patients with MT [8,17]. The finding of this study in terms of the rate of ICH following MT was in line with a systematic review by Marquardt et al. on patients from their center as well as previously reported case reports and cases series who underwent a reperfusion therapy following LVO due to IE, either MT or the administration of IVT [17]. In that study, the analysis was conducted on 40 individuals and showed that the rate of occurrence of any ICH was significantly higher in those with IVT (63%) compared with those with MT (18% with a 95% CI of 4% to 51%), which was close to the pooled rate in our study. The results of another systematic review also showed a more frequent occurrence in those under treatments with thrombolytics, either alone or in a combination [8].

This study had several limitations. Only a few studies with a relatively low level of evidence were found to be eligible to be considered in the final quantitative analysis. Contrary to the two previously published systematic reviews, we did not include case reports or case series with a small number of cases to have more generalizable conclusions; however, even with the newly published reports, the overall number of the included patients in this study was nearly triple their numbers. The low number of included studies also limited us when conducting the sub-group analyses; in particular, the outcome parameters in patients receiving different therapeutic strategies, IVT alone, MT alone, and a combination therapy. Last, although Egger’s test showed no publication bias in this study, the results warrant being used with caution.

## 5. Conclusions

Our study showed that MT could significantly decrease the extent of neurological deficits that patients experience due to strokes caused by LVO related to IE. In addition to this clinical effect, the rate of post-therapy complications such as ICH seems to be reasonably lower with this therapeutic strategy compared with IVT. Large-scale randomized clinical trials and prospective cohort studies are warranted.

## Figures and Tables

**Table 1:** Characteristics of the included articles investigating the safety and outcomes of MT for patients with strokes secondary to IE.

Author	Country	Design	Age, Mean (SD) or Median (IQR)	Sex (Female)	Follow-Up	*N.*	Pathology	Risk Factors (N.)	Occluded Vessel Side (Left)	Vessel (N.)	IVT (N.)
Feil, 2020 [15]	Germany	Cohort/ case-control	69 (13.3)	24	24 h	55		HTN: 37, DM: 10, hypercholesterolemia: 16, smoker (current/former): 14, and AF: 18	30	BA: 8, MCA M1: 32, MCA M2: 9, and other, including ACA, carotis-T, ICA intracranial, ICA extracranial, and VA: 11	8
					Three months						
Marnat, 2020 [16]	France	Case-control	59.2 (17.6)	8	24 h	28	*Enterococcus faecalis* (6/28), *Pseudomonas aeruginosa* (1/28), *Staphylococcus aureus* (1/28), *Staphylococcus capitis* (1/28), *Streptococcus viridans* (1/28), *Streptococcus gordonii* (1/28), unidentified streptococcus (1/28), *Candida parapsilosis* (1/28), *trichosporon* (1/28), unidentified Gram-negative bacillus (1/28), unidentified Gram-positive bacillus (1/28), and unidentified germs (12/28). Bacteriological–histological analysis of the thrombus allowed IE diagnosis in five cases	HTN: 9/27, DM: 3/27, hypercholesterolemia: 9/27, current smoker: 8/25, antithrombotic use: 17/27, coronary artery disease: 6/27, and previous stroke/TIA: 3/27		MCA M1: 16/28, MCA M2: 3/28, tandem: 1/28, intracranial ICA: 5/28, VB: 2/28, and extracranial ICA/others: 1/28	8
					Three months						
Marquardt, 2019 [17]	USA	Case series	57.5 (11.6)	0	24 h	4	*Bartonella quintaina*, *Streptococcus anginosus*, *Staphylococcus aureus*, and unknown		Left: 2	MCA M1: 4	1
Ramos, 2020 [13]	Spain	Case series	64.33 (14.25)	6	24 h	12	*Streptococcus viridans:* 1, methicillin-resistant *Staphylococcus epidermidis*: 1, methicillin-resistant *Staphylococcus aureus*: 3, *Enterococcus faecalis*: 3, *Staphylococcus epidermidis:* 1, *Klebsiella pneumoniae*: 1, and negative: 2	AF: 6, anticoagulant: 7, and surgery: 6		MCA M1: 9, MCA M2: 2, and intracranial ICA: 1	2
					Three months						
Sader, 2021 [18]	USA	Case series	59 (range 29–80)	5	Unknown	15	*Staphylococcus aureus*: 2, *Staphylococcus epidermidis*: 1, *Enterococcus faecalis*: 4, Alpha Strep: 1, *Streptococcus parasanguinis*: 1, *Streptococcus mitis*: 1, *Aspergillus fumigatus*: 1, *Candida tropicalis*: 1, *Streptococcus agalactiae*: 1, *Streptococcus salivarius*, *Staphylococcus hominis*, coag-negative *Staphylococcus*: 1, and unknown: 1		Left: 11, and right: 5	MCA M1: 12, MCA M2: 1, carotid terminus: 2, and cavernous: 1	6
Ambrosioni, 2018 [19]	Spain	Case series	75.5 years (interquartile range 59–79 years)	3	24 h	6	*Staphylococcus aureus*: 1, *Streptococcus oralis*: 1, *Streptococcus dysgalactie*: 1, *Staphylococcus epidermidis*: 1, and negative culture: 2			Sub-occlusion tandem (carotid plus M1): 1, MCA M1: 3, BA: 2	
					Seven days						
					Three months						

ACA: anterior communicating artery; AF: atrial fibrillation; BA: basilar artery; DM: diabetes mellitus; HTN: hypertension; IE: infective endocarditis; IVT: intravenous thrombolysis; MCA: middle cerebral artery; N: number of patients; VA: vertebral artery.

**Table 2:** Details and outcomes of included articles investigating the safety and outcomes of MT for patients with strokes secondary to IE.

Author	Follow-Up	Baseline NIHSS, Mean (SD)	Treatment Approach for Intracranial LVO	Periprocedural Complications	Reperfusion (TICI)	NIHSS in F/U Mean (SD)	ICH in F/U (N.)	Complications during Hospital Stay (N.)	Mortality or mRS = 6 (N.)	Recurrent Stroke (N.)
Feil, 2020 [15]	24 h	15 (8.14)	Aspiration catheter solo: 11, stent retriever solo: 10, combination: 31, and additive medication during MT: 13	Device malfunction: 2, dissection and perforation: 2, clot migration and embolization: 1, ICH: 3, vasospasm: 2, and other: 4	mTICI 2b/3: 41	16 (17.65)	14	Malignant MCA infarction: 7, recurrent stroke: 7, ICH: 17, groin hematoma: 2, groin aneurysm: 2, and other complications: 27		7
	Three months								33	
Marnat, 2020 [16]	24 h	16.5 (6.66)	Aspiration: 11/25, stent retriever: 6/25, combination: 4/25, and balloon + SR: 4/25	2	mTICI 3: 12/28, mTICI 2c/3: 20/28, and mTICI 2b/3: 24/28	−3 (6.66) *	Any ICH: 10, parenchymal hematoma: 2, and sICH: 2			7/28
	Three months								7/27	
Marquardt, 2019 [17]	24 h	17.25 (3.89)	MT solitaire and penumbra: 2, MT solitaire and wingspan stent: 1, and MT penumbra with 2 mg tPA: 1		TICI 2A: 2, TICI 2B: 1, andTICI 3: 1	14 (3.31)	2	ICH: 2		
Ramos, 2020 [13]	24 h	13.08 (6.94)		2	TICI 0: 3, TICI 1: 2, TICI 2A: 1, TICI 2B: 1, andTICI 3: 5	10.75 (8.92)	6	6	3	
	Three months									
Sader, 2021 [18]	Immediately after	17.93 (5.37)			TICI 0: 1, TICI 2a: 1, TICI 2c: 1, TICI 2b: 4, and TICI 3: 6	12.71 (8.59)	7		2	
	discharge					9.2 (6.57)				
Ambrosioni, 2018 [19]	24 h	14.33 (10.19)	Stent retriever plus carotid stent: 1, and stent retriever: 5		mTICI 0: 1, mTICI 2B: 1, and mTICI 3: 3	12.66 (15.8)	0			
	Seven days								2	
	Three months								3	

F/U: follow-up; ICH: intracranial hemorrhage; MCA: middle cerebral artery; MT: mechanical thrombectomy; mRS: modified Rankin Scale; N: number of patients; NIHSS: National Institute of Health Stroke Scale; SR: stent retriever; TICI: Thrombolysis in Cerebral Infarction; tPA: tissue plasminogen activator. * Change was reported.

## Data Availability

The data presented in this study are available in the original text and Supplementary Materials.

## Supplementary Materials

The following supporting information can be downloaded at: https://www.mdpi.com/article/10.3390/life12122146/s1. Part A: Search Strategy; Part B: Forest plots of ICH, symptomatic ICH, mortality and stroke recurrence, and their funnel plots; Part C: A more detailed table of outcomes of studies investigating the safety and outcome of MT for patients with stroke secondary to IE.
