# Supplementary material for: Endovascular Treatment of Large Vessel Occlusion Strokes Caused by Infective Endocarditis: A Systematic Review, Meta-Analysis, and Case Presentation
Source: Life (Basel). 2022 Dec 19;12(12):2146. doi: 10.3390/life12122146 (PMC9780851; doi:10.3390/life12122146)
Supplement: Supplementary file 1 [file life-12-02146-s001.zip › life-2051978-supplementary.pdf]

Supplementary Materials

Part A: Search Strategy.
Part B: Forest plots of ICH, mortality, recurrence, and funnel plots of analyzed studies.
Part C: A more detailed table of outcomes of studies investigating the safety and outcome of MT for patients with stroke secondary to IE.

Part A: Search Strategy

(((((Endocarditis[MeSH Terms]) OR (Infective Endocarditis[Title/Abstract])) OR (Infective Endocarditides[Title/Abstract])) OR (Septic emboli[Title/Abstract])) AND ((((((Stroke[MeSH Terms]) OR (Cerebrovascular Accident[Title/Abstract])) OR (CVA[Title/Abstract])) OR (Brain Vascular Accident[Title/Abstract])) OR (Brain infarct[Title/Abstract])) OR (Large vessel occlusion[Title/Abstract]))) AND (((((((((((((Thrombectomy[MeSH Terms]) OR (Mechanical Thrombolysis[MeSH Terms])) OR (Endovascular Procedures[MeSH Terms])) OR (Thrombectomies[Title/Abstract])) OR (Percutaneous Aspiration Thrombectomy[Title/Abstract])) OR (Percutaneous Aspiration Thrombectomies[Title/Abstract])) OR (Mechanical Thrombectomy[Title/Abstract])) OR (Mechanical Clot Disruption[Title/Abstract])) OR (Endovascular treatment[Title/Abstract])) OR (Endovascular Procedure[Title/Abstract])) OR (Intravascular Procedure[Title/Abstract])) OR (Intravascular Technique[Title/Abstract])) OR (Endovascular Technique[Title/Abstract])) OR (Endovascular[Title/Abstract]))

Part B: Forest plots of ICH, mortality, recurrence, and funnel plots of analyzed studies.

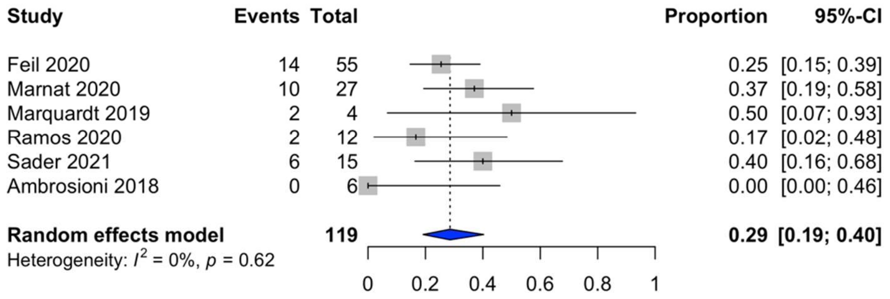

Figure S1. Forest plot of pooled rate of ICH.

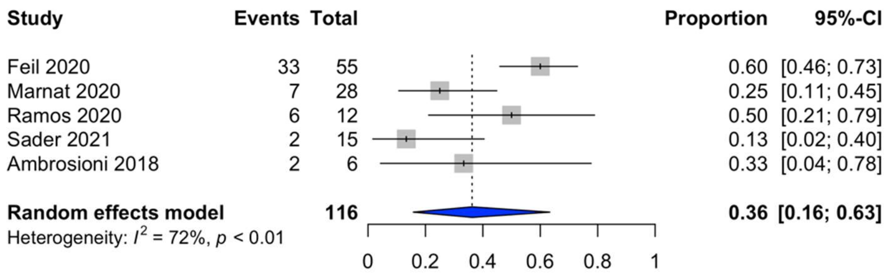

Figure S2. Forest plot of pooled rate of mortality.

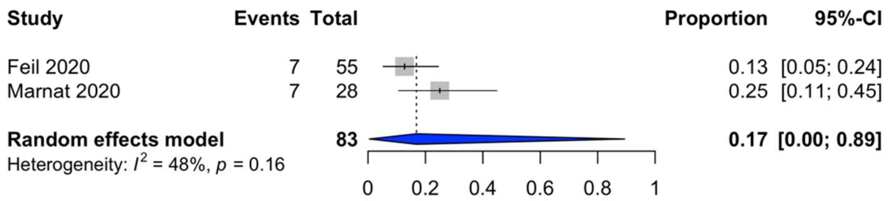

Figure S3. Forest plot of pooled rate of stroke recurrence.

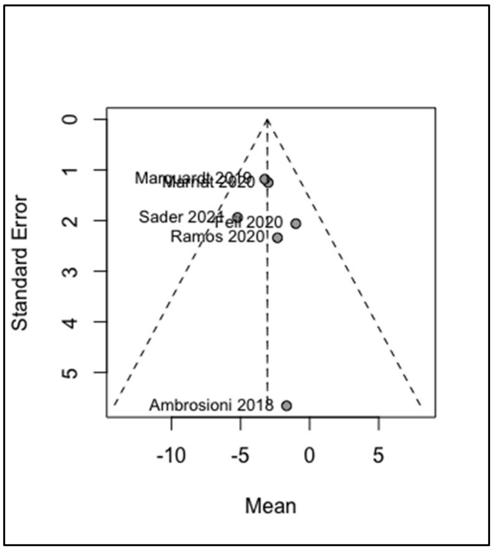

Figure S4. Funnel plot of pooled WMD of NIHSS.

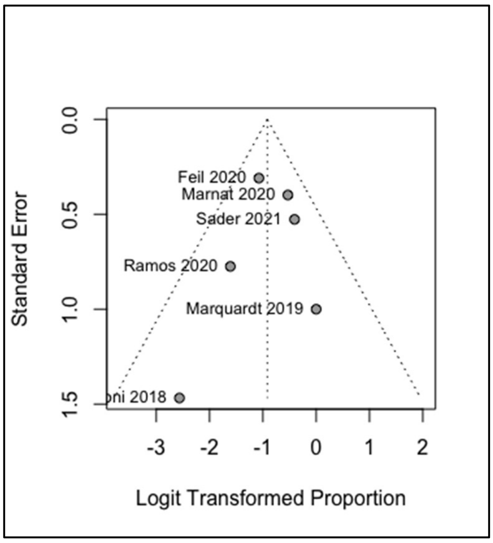

Figure S5. Funnel plot of pooled rate of ICH.

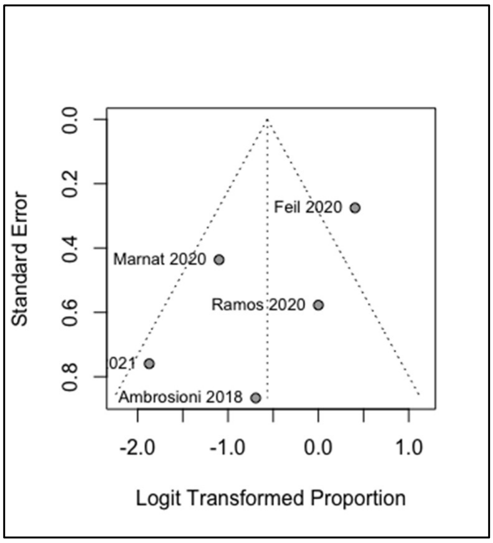

Figure S6. Funnel plot of pooled rate of mortality.

Part C: A more detailed table of outcomes of studies investigating the safety and outcome of MT for patients with stroke secondary to IE.

Author	Follow up	Baseline NIHSS	Baseline ASPECT	Baseline mRS	Treatment approach for intracranial LVO,	Periprocedural complications	reperfusion mTICI	NIHSS in F/U	ICH in F/U	Complications during hospital stay	Mortality, or mRS= 6
Feil 2020	24 hrs	15(8.14)	8(7-10)	1 (0–1) available in n = 54	Aspiration catheter solo: 11<br>Stent retriever solo: 10<br>Combination:31<br>Additive medication during MT: 13	Device malfunction : 2<br>Dissection, perforation: 2<br>Clot migration, embolization: 1<br>ICH:3<br>Vasospasm: 2<br>Other: 4	mTICI 2b/3: 41	16 (17.65)	14	Malignant MCA infarction: 7<br>Recurrent stroke: 7<br>ICH:17<br>Groin hematoma:2<br>Groin aneurysm:2<br>Other complications:27	
	3 months										33
Marnat 2020	24 hrs	16.5 (6.66)	7.0 (6.0 to 8.0)		Aspiration : 11/25<br>Stent retriever : 6/25<br>Combination:4/25<br>Balloon + SR: 4/25	2	mTICI 3:12/28<br>mTICI 2c/3: 20/28<br>mTICI 2b/3:24/28	- 3 (6.66) *	Any ICH:10<br>Paranchymal Hematoma:2<br>sICH:2		
	3 months										7/27
Marquardt 2019	24 hrs	17.25(3.89)			Mechanical thrombectomy - Solitaire and Penumbra :2<br>Mechanical thrombectomy-Solitaire and Wingspan stent: 1<br>Mechanical thrombectomy-Penumbra with 2mg IA-tPA:1		TICI 2A:2<br>TICI 2B:1<br>TICI 3:1	14(3.31)	2	ICH:2	
Ramos 2020	24 hrs	13.08(6.94)	9(8-10)			2	TICI 0:3<br>TICI 1:2<br>TICI 2A: 1<br>TICI 2B:1<br>TICI 3:5	10.75(8.92)	2	6	6
	3 months										
Sader 2021	UK	17.93(5.37)		0 (0-4)			TICI 0:1<br>TICI 2a:1<br>TICI 2c:1<br>TICI 2b:4<br>TICI 3:6	12.71(8.59)<br>9.2(6.57)	6		2
Ambrosioni 2018	24 hrs	14.33(10.19)			Stent retriever plus carotid stent : 1<br>Stent retriever:5		mTICI 0 :1<br>mTICI 2B: 1<br>mTICI 3: 3	12.66(15.8)	0		

	7 days										2
	3 months										3

F/U: Follow-up, ICH: Intracranial Hemorrhage, MCA: Middle Cerebral Artery, MT: Mechanical Thrombectomy, mRS: Modified Rankin Scale, N: Number of patients, NIHSS: National Institute of Health Stroke Scale, SR: Stent Retriever, TICI: Thrombolysis in Cerebral Infarction, tPA: Tissue Plasminogen Activator.

* Change has been reported.
